# Alzheimer’s Disease beyond Calcium Dysregulation: The Complex Interplay between Calmodulin, Calmodulin-Binding Proteins and Amyloid Beta from Disease Onset through Progression

**DOI:** 10.3390/cimb45080393

**Published:** 2023-07-27

**Authors:** Danton H. O’Day

**Affiliations:** 1 Department of Biology, University of Toronto Mississauga, Mississauga, ON L5L 1C6, Canada; danton.oday@utoronto.ca; 2 Cell and Systems Biology, University of Toronto, Toronto, ON M5S 3G5, Canada

**Keywords:** Alzheimer’s disease, calcium dysregulation, calmodulin, calmodulin-binding proteins, amyloid beta, ion channels, Tau, neuroinflammation, neurodegeneration

## Abstract

A multifactorial syndrome, Alzheimer’s disease is the main cause of dementia, but there is no existing therapy to prevent it or stop its progression. One of the earliest events of Alzheimer’s disease is the disruption of calcium homeostasis but that is just a prelude to the disease’s devastating impact. Calcium does not work alone but must interact with downstream cellular components of which the small regulatory protein calmodulin is central, if not primary. This review supports the idea that, due to calcium dyshomeostasis, calmodulin is a dominant regulatory protein that functions in all stages of Alzheimer’s disease, and these regulatory events are impacted by amyloid beta. Amyloid beta not only binds to and regulates calmodulin but also multiple calmodulin-binding proteins involved in Alzheimer’s. Together, they act on the regulation of calcium dyshomeostasis, neuroinflammation, amyloidogenesis, memory formation, neuronal plasticity and more. The complex interactions between calmodulin, its binding proteins and amyloid beta may explain why many therapies have failed or are doomed to failure unless they are considered.

## 1. Introduction

The primary cause of dementia in those over 65 years of age, Alzheimer’s disease (AD) currently affects just under 60 million people worldwide, with no evidence the incidence of the disease and its socioeconomic effects are abating [1]. As the major cause of dementia worldwide, AD is destined to significantly increase because of its age dependency. It is a complex, multifactorial disease, historically characterized by the organization of amyloid beta (Aβ) peptides into senile plaques and the phosphorylation of tau to generate neurofibrillary tangles with resulting neurodegenerative effects [1,2]. While there are clear genetic links to the early onset form of AD, late-onset AD is typically sporadic, with multiple factors involved, including risk genes, neuroinflammation, calcium dysregulation, mitochondrial dysfunction and reactive oxygen species (ROS). Coupled with this are lifestyle, gender and other factors. As the number of cases of AD continues to grow worldwide, no treatment exists to stop it, let alone significantly slow its interminable progress, but many new therapies are currently under study, and a new monoclonal anti-amyloid therapy has been introduced [3].

**Figure 1:**
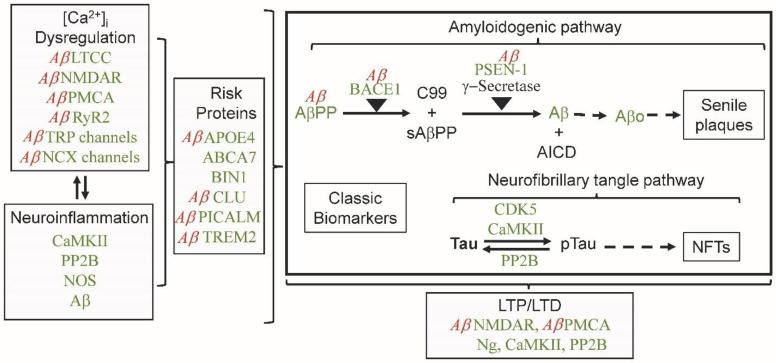
Some of the calmodulin-binding proteins (green text) and their interaction with amyloid beta (Aβ, red) during the onset and progression of Alzheimer’s disease. Neuroinflammation and calcium dysregulation are early, likely interacting, critical events. Their impact depends on other factors, such as the presence of risk proteins. Downstream symptoms of the disease are realized through the progressive accumulation of amyloid beta and phospho-Tau (pTau), culminating in the production of senile plaques and neurofibrillary tangles (NFTs). The generation of amyloid beta monomers and oligomers as well as the build-up of pTau are involved in events linked to the disruption of neurotransmission and synaptic dysfunction, underlying memory and cognitive changes. At each stage calmodulin-binding proteins have critical roles that, in many cases, are impacted by their interaction with amyloid beta monomers/oligomers as detailed in the text. Abbreviations: ABCA7, ATP-binding cassette subfamily A member 7; Aβ, amyloid beta; Aβo, amyloid beta oligomers AβPP, amyloid beta precursor protein; APOE4, apolipoprotein E4; BACE1, β-secretase; BIN1, bridging integrator 1; CaMKII, Ca^2+^/CaM-dependent protein kinase II; CLU clusterin; CDK5, cyclin-dependent kinase 5; LTCC, L-type calcium channels; NCX channels; sodium/calcium exchanger; NFTs, neurofibrillary tangles; NMDAR, N-methyl-D-aspartate receptor; NOS, nitric oxide synthase; PICALM, phosphatidylinositol-binding clathrin assembly protein; PMCA, plasma membrane calcium ATPase; PP2B, protein phosphatase 2B (calcineurin); PSEN-1, presenilin; RyR2, ryanodine receptor 2; TREM2, triggering receptor expressed on myeloid cells 2; TRP channels, transient receptor potential channels; Voltage-gated calcium channels (VGCC).

As a multifactorial disease with the temporal contribution of various interacting elements not completely clarified, this review is organized around the events covered in Figure 1, specifically focusing on the calmodulin (CaM)-binding proteins (CaMBPs; green text) that are involved and, where it occurs, their binding to and potential regulation by amyloid beta (red text).

## 2. Calcium Dysregulation and Calmodulin

The critical role of calcium homeostasis in neuronal functioning has long been realized [4,5]. Calcium signaling is fundamental to learning and memory, neurotransmitter synthesis and membrane excitability. Its disruption can impact each of these events. Maintaining intracellular calcium levels is a critical process that is mediated by a multitude of ion channels and calcium-binding proteins [6]. Since that early work, a number of new contributors to calcium homeostasis and their modes of regulation have been revealed, including buffers, calcium-binding proteins, channels, exchangers, pumps and transporters [7]. With its normal intracellular concentration tightly controlled within a range from 10^−7^ to 10^−8^ M, minor but persistent disruptions in calcium levels can be harmful. Evidence for dysregulated calcium levels in the early events of AD led to the Calcium Hypothesis [8,9,10]. The Calcium Hypothesis is based on the concept that an unregulated influx of calcium ions into neurons is an initiating event that results in the production of the classic hallmarks of AD as follows: amyloid beta plaques and neurofibrillary tangles (NFTs), which, in turn, drive neurodegeneration.

Research on AD brains and experimental models has shown Ca^2+^ dyshomeostasis occurs prior to symptoms suggesting it is an early event in AD pathogenesis. Calcium dysregulation and the production of the classic hallmarks of AD, Aβ and Tau and their aggregation, are intimately related [7,11]. The subsequent buildup of Aβ in the brains of AD sufferers causes the influx of Ca^2+^ from the extracellular space, augmenting Ca^2+^ levels that can, in turn, affect Aβ production, Tau hyperphosphorylation and NFT formation [12,13,14]. In their review article title, Webber et al. [15] state that intracellular calcium is the “Judge, jury and executioner of neurodegeneration” for AD. This is misleading because while calcium may be critical, it is not sufficient to cause neurodegeneration. It cannot work alone but instead operates through target binding, especially via Ca^2+^-binding proteins (CaBP), of which CaM, as detailed here, is arguably a main and primary target in normal and AD brain cells.

### 2.1. Calcium Regulation of Calmodulin

A primary target of neuronal and microglial calcium signaling, the calcium-binding protein CaM functions by binding to and regulating CaM-binding proteins (CaMBPs) [16]. CaM is expressed at micromolar concentrations in neurons, with the highest levels found in cortical regions, striatum, hippocampus, amygdala and substantia grisea [17]. A single immunological study suggests that CaM levels decrease in many of these regions in the AD brain [18]. This relatively small (148aa; 16.7 kDa), highly conserved calcium-binding protein can bind to CaMBPs either in the absence of calcium (apoCaM) state or when it is calcium-bound (Ca^2+^/CaM) [16,19,20,21]. Unlike other protein–protein binding, Ca^2+^/CaM can interact with a diversity of binding domains primarily defined by the positioning of hydrophobic amino acids within an 18–22 amino acid stretch anchored by acidic residues. Well over 300 calcium-dependent CaMBPs have been identified [22]. Calcium-dependent binding is divided into the following number of binding motif subclasses: *1-10 Subclasses*, 1-10, (FILVW)xxxxxxxx(FILVW)); 1-5-10, (FILVW)xxx(FAILVW)xxxx(FILVW); Basic 1-5-10, (RK)(RK)(RK)(FAILVW)xxx(FILV)xxxx(FILVW)); 1-12 Subclass, 1-12, (FILVW)xxxxxxxxxxXXxx(FILVW); 1-14 Subclasses, 1-14, (FILVW)xxxxxxxxxxxx(FILVW); 1-8-14, (FILVW)xxxxxx(FAILVW)xxxxx(FILVW); Basic1-8-14, (RK)(RK)(RK)(FILVW)xxxxxx(FAILVW)xxxxx(FILVW); 1-5-8-14, (FILVW)xxx(FAILVW)xx(FAILVW)xxxxx(FILVW); 1-16 Subclass, 1-16, (FILVW)xxxxxxxxxxxxxx(FILVW). Non-canonical binding has been less well studied but includes various hydrophobic amino acid arrangements, short sequences and/or myristoylated proteins. ApoCaM binding is mediated via IQ ([FILV]Qxxx[RK]Gxxx[RK]xx[FILVWY]), IQ-like ([FILV]Qxxx[RK]Gxxxxxxxx) motifs or other IQ variants. This binding domain flexibility underlies CaMs ability to bind and regulate a diversity of proteins that are linked to AD and other neurodegenerative diseases [23,24].

### 2.2. Calmodulin Hypothesis of Alzheimer’s Disease

Since CaM was shown to bind to and regulate CaMBPs critical to the formation of the hallmarks of AD (amyloid plaques and neurofibrillary tangles), the Calcium Hypothesis was extended to the Calmodulin Hypothesis [25]. Continued research strengthened support for the Calmodulin Hypothesis by revealing that CaM also binds to and regulates many risk factor proteins, metabotropic glutamate receptors (mGluR), ryanodine receptors, the adenosine A2A receptor and other proteins involved in the onset and progression of AD and other neurodegenerative diseases [23,26,27,28,29]. Critical events in neuroinflammation, including the activation of microglia, are also regulated by CaM [24,30].

### 2.3. Calmodulin Regulation of Calcium Homeostasis

**Figure 2:**
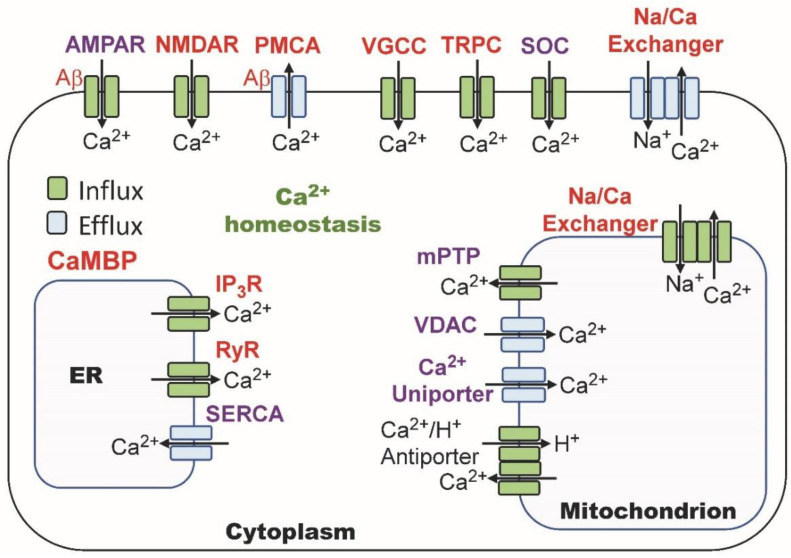
Some regulatory aspects of ion channels that are involved in calcium homeostasis. Calcium influx (green channels) into the cytoplasm occurs across the cell membrane through multiple channels (NMDAR, AMPAR, VGCC, SOC, TRPC). Cytoplasmic contributions to increased intracellular calcium levels come from the endoplasmic reticulum (ER; IP3R, RYR) and mitochondria (Na^+^/Ca^2+^ exchangers, Ca^2+^/H^+^ antiporter, mPTP). Intracellular calcium levels can be reduced by efflux (blue channels) via the cell membrane (Na^+^/Ca^2+^ exchangers, PMCA) or by uptake into the ER (SERCA) and mitochondria (Ca^2+^ uniporter, VDAC). A number of calcium ion channels bind to and are regulated by CaM (Red/bold text: NMDAR, VGCC, TRPC, IP_3_R, RyR), while others are regulated by the Ca^2+^/CaM-dependent protein kinase II (CaMKII) (Purple/bold text: AMPAR, PMCA, SOC, SERCA, mPTP, VDAC, Ca^2+^ uniporter). Two channels involved in calcium homeostasis also bind to amyloid beta (red text, AMPAR, NMDAR) as discussed below. Abbreviations: AMPAR, α-amino-3-hydroxy-5-methyl-4-isoxazoleproprionic acid receptor; Ca^2+^ uniporter, mitochondrial calcium uniporter; Ca^2+/H+^ antiporter, calcium/hydrogen ion exchanger; IP_3_R, inositol 1,4,5 trisphosphate receptor; LTCC, L-type calcium channels; mPTP, mitochondrial permeability transition pore; NCX channels; sodium/calcium exchanger; NMDAR, N-methyl-D-aspartate receptor; PMCA, plasma membrane calcium ATPase; SERCA, sarco/endoplasmic reticulum Ca^2+^-ATPase; SOC, store-operated calcium channel; RyR2, ryanodine receptor 2; TRPC, transient receptor potential channels; VDAC, voltage-dependent anion channel; Voltage-gated calcium channels (VGCC).

CaM is also involved in regulating calcium homeostasis. Over a dozen ion channels located in different areas (cell membrane, mitochondria, endoplasmic reticulum) maintain intracellular calcium levels (Figure 2). Of interest here is the role of CaM and Aβ in regulating specific ion channels. The following multiple calcium ion channels bind to and are regulated by CaM: NMDAR, PMCA, VGCC, TRPC, Na/Ca Exchangers, IP3R and RYR. As will be discussed, AMPAR is not a CaMBP, but it is regulated by two CaMBPs (CaMKII and PP2B) as well as Aβ. The CaMBP NMDAR also has an indirect interaction with Aβ, which is also covered below (Section 6.1).

Other channels that are regulated by CaM have been well studied. Voltage-gated calcium channels (VGCC, Ca_v_) initiate synaptic transmission in presynaptic neurons and play a central role in calcium signaling events central to models of learning, memory and plasticity [31,32]. Calcium influx through Cav2.1 and Cav2.2 channels initiates synaptic vesicle exocytosis [33]. VGCCs comprise 10 subfamilies (L-type (CaV1.1, CaV1.2, CaV1.3, CaV1.4), P/Q-type (CaV2.1), N-type (CaV2.2) and R-type (CaV2.3)) that are regulated by CaM through C-terminal IQ motifs ([I/L/V]QXXXRXXXX(R/K) [31,34,35]. Among other things, the influx of the divalent cations is regulated by calcium-dependent inactivation (CDI) that can be eliminated by IQ-specific mutations [36]. As covered below (Section 6.1), CDI was recently demonstrated in NMDAR [37].

Various other CaMBPs that are not ion channels are also involved in maintaining calcium homeostasis. The calcium levels in the ER are monitored by stromal interaction molecule (STIM) proteins, which interact with the Orai1 voltage-independent calcium channel to permit calcium influx as needed [38]. The following two STIM species exist in hippocampal neurons: STIM1 is involved in calcium regulation in the developing neuron, while STIM2 carries out this function in the adult neuron [39]. STIM2 (Stromal interaction molecule 2) is a multifunctional resident ER protein involved in regulating calcium levels in the ER and cytoplasm via store-operated calcium entry (SOCE) [40,41,42]. Colocalizing with CaMKII in hippocampal mature (mushroom) spines, STIM2 is involved in LTP and post-synaptic plasticity [40,43]. Two of the three STIM2 isoforms (STIM2.1, STIM2.2) possess a CaM-binding domain in their C-term, the region where interactions with other proteins involved in calcium regulation occur (e.g., STIM1, CRAC, Orai and TRPC). For example, CaM binding to STIM1 inhibits Orai1 binding, causing the closing of the Orai1 calcium channel [44]. Several TRPC isoforms that have been evaluated as AD therapeutic targets are also regulated by CaM [45,46]. Clearly, the whole story on the regulation of calcium homeostasis and dysregulation by CaM is only coming to light.

## 3. CaMBPs and Neuroinflammation

The events of calcium dysregulation and neuroinflammation are intertwined and there is accumulating evidence that they occur early in most, if not all, neurodegenerative diseases prior to the appearance of biomarkers specific to the disease. By extension, the resulting disease process would then be dictated through the mediation of disease-specific risk factors and the activation of risk genes. The events and causes of neuroinflammation and their relationship to neurodegenerative diseases, such as AD and others, have been well-reviewed [47,48]. Neuroinflammation is an early and critical event in Alzheimer’s disease (AD), amyotrophic lateral sclerosis (ALS), frontotemporal dementia (FTD), Huntington’s disease (HD), Parkinson’s disease (PD), Lewy Body dementia (LBD), Batten disease (BD), traumatic brain injury (TBI) and others [49,50,51,52,53]. Mediated mainly by brain microglia and astrocytes and involving cross-talk with neurons, neuroinflammation is a complex, multistage process that, when unchecked, can lead to the uncontrolled release of proinflammatory factors, primarily from microglia that weaken synaptic function, impact neuronal repair and disrupt the blood–brain barrier (e.g., [47,48]). In AD, Aβ interaction with microglial surface receptors leads to an inflammatory response that, through the release of cytokines and other mediators, causes an increase in extracellular glutamate. The high levels of this neurotransmitter activate extrasynaptic GluN2B NMDARs and AMPAR, inducing LTP impairment through CaMKII inhibition and LTD enhancement via PP2B enhancement [54]. The following two main types of microglial cells exist: resting or quiescent and active [48]. While active microglia display a great deal of heterogeneity, they can be grouped into the following two opposite phenotypes, each with specific associated factors: M1 or proinflammatory (e.g., proinflammatory cytokines, nitric oxide (NO)) and M2 or anti-inflammatory (e.g., anti-inflammatory cytokines, neurotrophic factors). Proinflammatory events involve inducible NO synthase, which is involved in NO generation [55]. Inducible nitric oxide synthase is an experimentally verified CaMBP with CaM-binding leading to enzyme activation [56]. Aβ diminishes NOS activity indirectly by binding to NADPH and restricting its interaction with the enzyme [57].

In addition to iNOS, the following 11 other AD neuroinflammatory proteins are potential CaMBPs due to the presence of one or more CaM-binding domains (CaMBDs) with canonical calcium-dependent binding motifs: ABCA1 (ATP-binding cassette subfamily A member 1), ABCA7 (ATP-binding cassette subfamily A member 7), CD33 (myeloid cell surface antigen CD33), CH3L1/YKL-40 (chitinase-3-like protein I), CLU (clusterin), CR1 (complement receptor type 1), EPHA1 (ephrin type-A receptor 1), MS4A (membrane-spanning 4-domains subfamily A), NLRP3 (NACHT, LRR and PYD domains-containing protein 3), PILRA (paired immunoglobin-like type 2 receptor alpha) and TREM2 (triggering receptor expressed on myeloid cells 2) [24]. Of these, CLU and TREM2 are Aβ receptors [58,59]. In total, the results reveal that CaM plays multiple central roles in neuroinflammation.

## 4. CaM, CaMBPs and Aβ in Amyloidogenesis

Attempting to understand the changes in the brain during aging and neurodegenerative diseases is extremely challenging. While the classically defined AD neuropathological agents, Aβ and pTau, are diagnostic for AD and widely considered as driving forces in neurodegeneration, up to a third of individuals with Aβ/pTau pathology are cognitively normal [60,61]. On the other hand, some people show significant cognitive impairment in the absence of AD pathology or any other neural or cognitive abnormalities [62]. While Aβ is not a constant in neurodegenerative AD events, it is clearly a major factor in them, as will be clear from the information that follows.

Over 100 identified Aβ/Aβo receptors (hereafter, Aβ receptors) exist in the human brain, dozens of which are involved in neuroinflammation, calcium regulation and other critical events linked to neurodegenerative diseases [59,63,64,65]. Experimentally proven CaMBPs involved in AD that directly bind to Aβ include AβPP1, mGluR, NMDAR, PMCA and PSEN1 [66]. In addition, several AD risk factor proteins that are potential CaMBPs, and which also bind Aβ are TREM2 (triggering receptor expressed on myeloid cells 2), CLU/ApoJ, PICALM and three APOE isoforms (APOE 2-4) [59,67]. CaM is one of the cellular targets with the highest affinity for neurotoxic Aβ peptides. Aβ binds with a high affinity to CaM through the neurotoxic Aβ25-35 domain, and the affinity of Aβ for Ca^2+^/CaM is approximately 20-fold higher than for apoCaM [27]. With CaM as the major binding protein for Aβ, it follows that it could play a critical function in AD. The interplay between CaM and CaMBPs that bind Aβ is quite complex and may have significant implications for both the understanding of the role of this hallmark in AD and why therapies focusing upon it have not been successful [66].

CaM mediates the amyloidogenic pathway in a diversity of ways leading to a complex interaction that has just recently been evaluated [66]. CaM not only binds to and regulates key enzymes in the generation of amyloid beta, it also binds to and affects the behavior of Aβ itself. This interaction begins with the first processing steps of amyloid beta precursor protein (AβPP). AβPP binds to CaM, and treatment with CaM antagonists, such as W7, promotes its processing along the non-amyloidogenic pathway [68]. The disintegrin and metalloproteinase domain-containing protein ADAM10 is a major protease that directs APP processing away from the amyloidogenic pathway, and its activation results in decreased Aβ production, providing a protective function against AD [62]. BACE1 and ADAM10 physically interact in part via the N-term of BACE1 (residues 43–94) [68,69]. This region contains a calcium-dependent CaMBD [70]. This suggests that the BACE1/ADAM10 interaction may be regulated by CaM. Could this interaction be a controlling factor in the regulation of amyloidogenesis? ADAM10, a proven CaMBP, also binds CaM via a calcium-independent IQ-binding motif (IQQPPRQRPRE) [71]. Inhibition of CaM binding with CaM antagonists activates this protease. Interestingly, a number of natural compounds that increase ADAM10 expression are listed in Figure 3 of Manzine et al. [71]. They include several phytochemicals that are proven CaM antagonists (e.g., curcumin, resveratrol, quercetin), thus offering multiple CaM-based therapeutic agents for further study [72].

### BACE1 Regulation by CaM and Aβ

As the initial enzyme in the amyloidogenic pathway, BACE1 has been well studied. In the presence of CaM, BACE1 activity is increased 2.5-fold in vitro [70]. In early onset and late-onset forms of AD, Aβ provides a positive feedback loop in its own production. Increased Aβ levels activate BACE1 gene transcription that, in turn, increases the amount of BACE1 enzymes, resulting in increased amounts of Aβ [65,73]. Aβ increases both BACE1 and AβPP levels via DNA Aβ-interacting domains (AβID) in the AβPP and BACE1 promoters resulting in a feedback loop that ultimately increases Aβ production [74]. Via its binding to both apoCaM and Ca^2+^/CaM, AβPP and PSEN1, Aβ also feeds back on its own production [27,75,76]. While many details of these interactions remain to be elucidated, some insight is being gained. Transmembrane domain 1 (TMD1) of PSEN1 is the region that modulates Aβ generation. Aβ42 binds to TMD1, with resulting effects on Aβ generation [76]. Aβ levels are also depleted as they oligomerize on their road to forming plaques. Soluble Aβ oligomers can re-release Aβ monomers. In addition, the Aβ-CaM interaction slowdowns Aβ fibrillation. During early amyloidogenesis, it follows then that Aβ-CaM levels would increase as CaM sequesters the toxic peptide, but once saturation occurs, the Aβ levels would increase, and fibrillation would progress unabated. Thus, Aβ fibrillation appears to be a multistage back-and-forth event that might offer routes to therapeutic intervention.

Continued processing of AβPP after BACE1 is also mediated by CaM since the CaMBP PSEN-1, a component γ-secretase, catalyzes the release of Aβ, of which Aβ42, a peptide of 42 amino acids, appears to be the most toxic [77]. In addition to these experimentally validated CaMBPs, many putative CaMBPs (e.g., nicastrin, presenilin enhancer protein 2 (PEN-2) and presenilin-stabilizing factor APH-1) involved in amyloidogenesis have been shown to possess CaM-binding domains with appropriate binding motifs that remain to be experimentally validated [23,26]. These multiple interactions reveal that the amyloidogenic pathway story in AD is far from complete and that CaM and Aβ lie at the heart of this critical stage in the disease.

## 5. Tau Phosphorylation

The second classic hallmark of AD is NFT formation. Tau phosphorylation disengages it from microtubules, setting the stage for its polymerization into NFTs. CaM has at least four separate roles in this process. First, Tau itself binds to Ca^2+^/CaM, which prevents it from binding to microtubules [34,78]. Second, the following two CaM-dependent kinases can phosphorylate Tau: Ca^2+^/CaM-dependent protein kinase II (CaMKII) and cyclin-dependent kinase 5 (CDK5) [79,80]. Third, while its role in pTau dephosphorylation appears to be limited, PP2B is a Ca^2+^/CaM-dependent protein phosphatase that can dephosphorylate pTau [81]. Fourth, Tau binds to each of the PP2B subunits, an event that is inhibited by CaM [82]. Thus, CaM is intimately associated with the initial events in NFT production. That said, the significance of this involvement remains to be studied. On the other hand, there seems to be no evidence for the additional regulatory role of amyloid beta in NFT formation except via its direct binding to CaM, which could affect the activation of CaMKII, CDK5 and PP2B.

## 6. CaM, Aβ, LTP and LTD

AD involves a progressive decline in memory, thinking and reasoning, coupled with behavioral changes and diminishing social skills. The events of cognition, learning and plasticity and their changes during AD involve not only alterations in the signaling pathways but changes to the synaptic structure as well. Neural plasticity refers to the ability of neural networks to change functionally via growth and/or reorganization. Our slowly emerging understanding of these events boils down to a collection of receptors, ions and signaling proteins that have been primarily studied in the CA1 pyramidal neurons of the hippocampus in human and animal systems. Long-term potentiation (LTP) is the cellular analog of learning and memory [83]. With AD, decreases in glutamate synthesis, calcium dysregulation plus the loss of both synapses and ion channels cause a failure of LTP, effectively resulting in memory loss. Hayashi [83] reviewed the history of LTP from its electrophysiological origin to its current cellular signaling and molecular underpinnings. There are different types of LTP, but the NMDAR/AMPAR-mediated type is the most common and functionally relevant and has been most studied in CA1 neurons of hippocampal pyramidal cells [84]. The basic series of events of LTP and its counterpart long-term depression (LTP) that are mediated by CaM and Aβ is the focus here.

LTP and LTD are types of synaptic plasticity. The balance between them is critical to learning and memory [83,85]. Rather than being opposing events, LTP and LTD work together to acquire and store information, respectively [86]. Depending on the length and intensity of calcium fluxes in dendritic spines, Ca^2+^/CaM can either activate CaMKII, driving LTP, or it can activate CaN, leading to LTD [81,87,88]. The regulation of CaMKII and PP2B by CaM has been extensively reviewed [89,90]. Differential calcium influxes can have these opposite effects due to the different CaM-affinity of CaMKII vs PP2B, coupled with their different spatial distribution within the synapse, as covered below (Section 6.3). While many issues remain to be resolved, the following four key players in LTP and LTD are CaMBPs: GluN2B NMDARs, CaMKII, PP2B. While not a CaMBP, AMPAR is regulated by CaMKII and PP2B.

### 6.1. NMDAR: A CaM-Binding Ion Channel

NMDARs are heterotrimers consisting of two GluN1 subunits plus two GluN2 or GluN3 subunits [91]. Abnormal NMDAR activity has been linked to Alzheimer’s and other neurodegenerative diseases. Because of its central role in critical neuronal functions and plasticity, NMDAR is under tight control, with much of the regulation involving CaM. CaM operates intracellularly in both fast and slow receptor activities [37]. During fast (millisecond) regulation, calcium influx reduces the open rate and time of the NMDAR ion channels as a result of Ca^2+^/CaM binding to both the C0 and C1 cytoplasmic domains of the NR1 subunit, leading to calcium-dependent inactivation (CDI) [92]. On the other hand, slow (minutes) regulation involves alterations in the phosphorylation state by CaM-dependent enzymes (CaMKIIα, PP2B) and non-CaM-dependent kinases (PKA, PKC). The intracellular calcium ion concentration is maintained around 100 nM, but the ligand-activated opening of NMDAR channels can result in local micromolar levels [93]. To prevent cytotoxicity, CDI inhibits further calcium influx through NMDAR [37].

CDI is a widely used ion-channel regulatory mechanism that involves both apoCaM and Ca^2+^/CaM binding to cytoplasmic domains close to the channel pore opening [36,37]. During normal calcium homeostasis, apoCaM binds to an IQ-like domain in the C-terminal of NMDAR, enhancing channel opening [94]. When intracellular calcium levels rise, the resident apoCaM is transformed to Ca^2+^/CaM, leading to its repositioning at the Ca^2+^-dependent CaMBD. This results in the CDI of the ion channel. The C1 region CaMBD has a rare 1-7 calcium-dependent binding motif, found also in MARCKs (myristoylated, alanine-rich, C-kinase substrate) [95]. Evidence indicates apoCaM is bound to the C0, positioning it for a rapid response to an increase in calcium ions. The IQ motif present within the CaMBD (residues 841–865) in the C0 domain of the NR1 subunit has recently been analyzed (MQLAFAAVNVWRKNLQDR) and found to share conserved hydrophobic residues with the CDI-based IQ motif of CaV1.1/1.2 [96]. High levels of neurogranin (Ng) can also dissociate calcium from Ca^2+^/CaM, causing a local increase in apoCaM [97,98]. Dissecting CDI in the NMDAR has been difficult due to the number of ion channels (Figure 2) that contribute to calcium levels at the synapse and to other factors [37,96].

There is extensive evidence that amyloid beta plays a role in the loss of cognition associated with AD. Aβ inhibits LTP in human hippocampal neurons while enhancing LTD [54]. In APP transgenic mouse models, Aβo blocks LTP both in vivo and in vitro prior to amyloid plaque formation. Aβ immunotherapy protects against cognitive deficits in the mouse model while also stopping the Aβ-induced LTP impairment [54]. In keeping with this, Aβ and CaM are involved in regulating LTP/LTD at multiple levels. NMDAR is both a CaMBP and an Aβ receptor that is also regulated by the classic CaMBP CaMKII. There is an interplay between NMDAR and Aβ. Elevated levels of the toxic peptide disrupt NMDAR, altering Ca^2+^ homeostasis, in turn interfering with LTP induction and causing cognitive defects [99,100,101,102].

### 6.2. AMPAR: Ion Channel Regulated by CaMBPs

The central role of CaM in learning and memory also involves AMPARs. While CaM does not bind directly to AMPAR, it does bind to and regulate CaMBPs involved in the receptor’s translocation, membrane localization and function. AMPAR recycling is involved in LTP, neural plasticity and the cognitive decline of AD [103]. The presence of AMPAR in the post-synaptic membrane is evidence for LTP [103,104]. CaMKIIα and protein kinase Mζ (PKMζ) assist in maintaining LTP by phosphorylating AMPAR to enhance its membrane localization and by preventing its endocytosis [105]. AMPARs are recruited to the synapse to increase synaptic strength partly by assisting in the removal of the magnesium ion blockage of NMDARs [106]. AMPAR recruitment is a multistep process. It involves intracellular trafficking and exocytosis of the receptors either extrasynaptically or synaptically, after which they are organized into the PSD by various scaffolding proteins, some of which are phosphorylated by CaMKIIα [84,107,108]. Proteins involved in AMPAR synaptic clustering include the following: synaptic cell adhesion molecules (CAMs), neurexin, LRRTM (Leucine-rich repeat transmembrane neuronal protein 1), TARP and PSD-95. Of these, PSD-95 not only binds CaM but it is also regulated by CaMKIIα [109]. PSD-95 stabilizes the ionotropic glutamate receptors, AMPAR and NMDAR, at the synapse and couples them to signaling components [110]. PSD-95 also protects synapses from damage by Aβ [111].

CaMKIIα phosphorylates Stargazin, increasing its binding to PSD95 and trapping it in stragazin/PSD95/NMDAR synaptic complexes [112]. To add to this, CaMKII regulates PSD95 trafficking out of the spines by phosphorylating it (pSer73), thereby blocking LTP and spine growth [113]. An early event in AD is the loss of synapses, an event that is linked to increased levels of Aβ that cause synaptic depression, a decrease in spine density and a drop in PSD-95 levels [114,115]. At high concentrations of soluble oligomeric Aβ, AMPA receptors are constantly removed from the post-synaptic membrane by endocytosis, also promoting LTD over LTP [116]. Ca^2+^/CaM binding to PSD-95 also results in its removal from the PSD, along with bound AMPAR [109]. These are just some of the multiple interactions CaM, CaMKIIα and Aβ regulate during these events. It should also be remembered that Aβ binds CaM and, as a result, can impact the activity of CaMKII and other CaM-dependent proteins.

### 6.3. The Interaction between Neurogranin, CaMKII and PP2B

The neuron-specific, post-synaptic CaMBP neurogranin (Ng, also called RC3, p17, BICKS) is expressed at high levels in dendritic spines in the hippocampus and cerebral cortex, where it functions in synaptic plasticity and learning [117,118]. Synaptic dysfunction and loss are associated with cognitive loss, a central event in the progression of AD [115,119]. CaM-binding activates CaMKII, after which autophosphorylation results in a stable, calcium-independent enzyme. Activated, phosphorylated CaMKIIα (pT286) is involved in dendritic spine stabilization, LTP and memory formation. On the other hand, PP2B can dephosphorylate CaMKIIα causing destabilized spines, LDP and impaired memory formation. The dephosphorylation of CaMKIIα (pT286) at AD synapses is directly related to the severity of AD [120]. Along with impaired cognitive function, individuals diagnosed with MCI and AD show decreased levels and a redistribution of pCaMKIIα.

Part of the regulation of CaMKII and PP2B involves the well-studied CaMBP Ng that possesses an IQ motif (33IQASFRGHMARKKI46), allowing it to bind apoCaM [98]. Ng is also recognized as a biomarker for AD. Correlating with AD progression, Ng hippocampal levels decrease and their dendritic localization diminishes as CSF levels increase [121,122,123]. In AD patients, a decrease in brain Ng levels and its increase in cerebrospinal fluid has been linked to poor cognitive performance [124]. Animal models have shown that the knockdown of Ng inhibits LTP and impacts cognitive function while increasing levels promotes LTP and improves cognition [125]. Ng is concentrated in the dendritic spines of hippocampal neurons, where it sequesters CaM to control local Ca^2+^/CaM signaling events [126,127,128]. As a result, it functions in the regulation of long-term potentiation (LTP) and long-term depression (LTD) [88,104].

**Figure 3:**
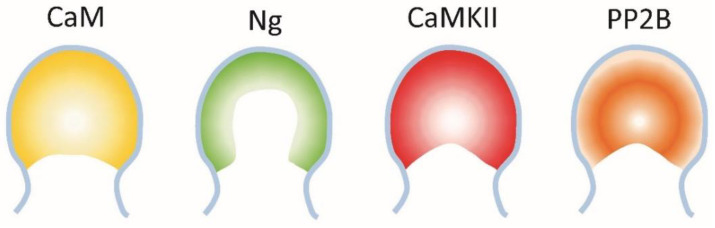
Localization of CaM, neurogranin (Ng), CaMKII and PP2B in synaptic boutons. Image after Zhong and Gerges [96].

CaMKII responds to high levels of calcium ions, while calcineurin is activated at lower levels. Calmodulin availability and activation by calcium ions thus is critical to both LTP and LTD. The binding of Ng to apoCaM allows it to localize, concentrate and control the accessibility of CaM at the synaptic membrane [88,96,129,130]. By sequestering calmodulin in rat hippocampal neurons, neurogranin lowers the threshold for LTP while increasing it for LTD [131]. The effect is not due to a shift in the localization of CaMKII or calcineurin. Instead, CaMKII sits adjacent to the cell membrane in dendritic spines, while calcineurin is localized away from it (Figure 3). Neurogranin localizes calmodulin near the cell membrane in closer proximity to CaMKII than calcineurin, which has implications for its role in LTP, LTD and synaptic plasticity.

## 7. Calmodulin and Synaptic Vesicle Exocytosis

While extensive evidence supports the decreased release of neurotransmitters as a critical event for the cognitive defects of AD, the impact of synaptic vesicle exocytosis is rarely covered. The synapse is the region where a presynaptic neuron interacts with a post-synaptic neuron. At a chemical synapse, a neurotransmitter is synthesized, packaged into exocytotic vesicles in presynaptic neurons and released by exocytosis in response to nerve stimulation. The diffusion of neurotransmitters across the synaptic gap sets them up for binding to post-synaptic receptors that, in response, initiate neuron-specific signaling pathways. While the majority of studies on hippocampal LTP/LDP focus on post-synaptic events, none of those could occur without the controlled presynaptic production and release of the neurotransmitter glutamate. Learning and memory, as exemplified by LTP and LTD, begin with the release of glutamate at synapses.

The stages of neurotransmitter exocytosis (vesicle trafficking, docking and vesicle fusion with the neuron cell membrane) involve complementary interactions between the following two calcium-binding proteins: calmodulin (CaM) and synaptotagmin [132]. During exocytosis, CaM binds to and regulates numerous regulatory proteins (e.g., VAMP, myosin V, Munc13, synapsin, GAP43, Rab3) as well as Ca^2+^/CaMKII that phosphorylates other exocytotic regulators (e.g., syntaxin, synapsin, RIM, Ca channels), thus playing a primary role presynaptically. Xue et al. [132] detail the regulation of these proteins and their roles in exocytosis. In keeping with the interplay between CaMBPs and Aβ in AD, Aβo inhibits exocytosis by inhibiting SNARE complex formation, possibly contributing to AD-impaired synaptic events [133]. As a result, CaM is a central functionary in the release of glutamate, the initiating neurotransmitter involved in learning and memory. As discussed above (Section 6.2), the recycling of the AMPAR back to the cell membrane of post-synaptic neurons involves the same exocytotic players [84].

## 8. Final Comments

Recent reviews provide insight into the significance of calcium dysregulation in AD and the underlying causes and effects of it as well as some potential therapies [15,134]. It is important to note that despite the central role of calcium, it requires other molecules that do its work. Calcium is essential but not sufficient to drive AD; it requires effectors such as CaM. While CaM is also essential, it too is not sufficient since it also needs effectors (CaMBPs) to carry out the functions it regulates. Sadly, except for passing references to two central CaMBPs (CaMKII and CaN), those reviews and others fail to address the fact that many of the primary calcium targets bind to and are regulated by CaM. What is more, as covered in this review, there are complex interactions between CaM, certain CaMBPs and Aβ that occur throughout the onset and progression of the disease.

Focusing on amyloidogenesis as a therapeutic target in Alzheimer’s disease has been unsuccessful, full of controversy and contradictory evidence, yet, for many valid reasons, it still dominates research and drug development strategies. In 2023, 26 of 187 ongoing drug trials are focusing on various aspects of the amyloid hypothesis, with many designed to eliminate or significantly diminish levels of Aβ monomers and/or oligomers and plaque load [3]. Regardless of the role of Aβ in AD, there are many problems with these strategies. Aβ is central to a number of essential biological functions [135,136,137]. It binds to a diversity of ion channels, neuronal receptors and other critical proteins. As reviewed here, some of these Aβ receptors bind to and/or are regulated by CaM, a primary calcium sensor and effector in neurons. For example, at least eight neuronal calcium ion channels are CaMBPs, at least two of which also are Aβ receptors. Aβ, acting as a transcription factor, feeds back to increase the production of critical proteins in the amyloid pathway. CaM itself has been shown to bind to and be regulated by Aβ. It is also clear that it and Aβ co-regulate major steps in LTP and LDP, not only in the regulation of the Ion channels themselves but in their channel operation and co-localization at the PSD. Until we understand these complex interactions between Aβ, CaM and its binding proteins at all stages of AD, simply trying to eliminate Aβ or any other single molecule will likely continue to lead to failure.

CaM exists at micromolar levels in neurons and, with a dissociation constant of approximately 1 nM, has a high affinity for Aβ interaction, adding another level of regulation to calcium signaling during AD. Poejo et al. [65] argue that CaM acts to sequester Aβ until the calcium-binding protein becomes saturated, after which Aβ levels will rise rapidly. Alternatively, by reducing the pool of available CaM, this interaction may be a way of limiting the function of CaM and its regulation of CaMBPs that are required for normal cellular functions [29]. CaM bound to Aβ1-42 (molar ratio 1:1) guards against Aβ fibrillogenesis [27]. This suggests a relevant, and up to now overlooked, direct neuroprotective role of CaM against Aβ neurotoxicity in the brain. On the other hand, the Aβ/CaM interaction could slow the availability of Aβ for binding to its receptors. A related issue is whether the Aβ binding to receptor biomarkers (e.g., CaM, Tau) masks immunological or other quantification, thus generating erroneous early-stage levels of Aβ, CaM or Tau. As a result, the data from an early, single report suggesting that CaM levels become reduced in critical areas of the AD brain is difficult to evaluate [18].

Despite the ongoing evidence for calmodulin and its binding proteins and their interactions with Aβ, not one current drug trial focuses on this area [3]. Even major CaMBPs that have been proven to be central to critical events in AD (e.g., CaMKIIα, PP2B) are apparently not targets of ongoing drug trials even though existing FDA-approved pharmaceuticals that target them already exist. Identifying critical CaMBPs involved in AD could provide more precise therapeutic targets than those directed at calcium. Many approved pharmaceuticals and phytochemicals exist that target CaM and specific CaMBPs that could be employed quickly as AD therapeutics [23,72].

While many pharmaceuticals exist that target CaM and certain CaMBPs, like any drug, they have side effects, which ongoing research is working to overcome. For example, the two classic CaMBPs that are central to critical events in AD, as covered above, CaMKII and PP2B, are classic examples that have been extensively studied. A large number of drugs have been developed that inhibit CaMKII function, and many more are under development [138]. KN-93 is an allosteric inhibitor of CaM binding targeting CaMKII in the inactive state, while AS105, GS-680 and RA306 are ATP-competitive inhibitors that inhibit the activated catalytic domain of CaMKII. These agents have side effects, including arrhythmia, ischemia, myocardial infarction and hypertension. Peptide inhibitors (e.g., CN19o isolated from the inhibitor CaMKIItide) are also being developed but, while less toxic, face issues with delivery and bioavailability. As new PP2B inhibitors are being developed, cyclosporin A and tacrolimus (FK506) are currently the most effective drugs in clinical application [139]. As with most pharmaceuticals, these two have side effects. including nephrotoxicity, neurotoxicity and hypertension.

## Data Availability

All of the data is in cited references to this review.
